# Measurements of Night Sky Brightness in the Veneto Region of Italy: Sky Quality Meter Network Results and Differential Photometry by Digital Single Lens Reflex

**DOI:** 10.3390/jimaging5050056

**Published:** 2019-05-24

**Authors:** Andrea Bertolo, Renata Binotto, Sergio Ortolani, Simone Sapienza

**Affiliations:** 1 Regional Environmental Protection Agency of Veneto, Via Ospedale Civile 24, 35121 Padova, Italy; 2 Department of Physics and Astronomy, University of Padova, vicolo dell’Osservatorio 2, 35122 Padova, Italy; 3 INAF, Osservatorio Astronomico di Padova, Vicolo dell’Osservatorio 5, 35122 Padova, Italy

**Keywords:** Night Sky Brightness, SMQ Network, Light Pollution, Differential Photometry

## Abstract

In this paper, we present the implementation of a monitoring network for artificial light at night (ALAN), based on Sky Quality Meter devices (SQM) installed in seven locations of the Veneto region. The system is coordinated by the Regional Environmental Protection Agency (ARPA-Veneto) and the Department of Physics and Astronomy of the University of Padova, in collaboration with a local dark-sky association, Venetostellato. A new centralized database containing zenith night sky brightness (NSB) data was implemented to collect data from all SQM stations of the regional territory, not only in real time (since 2017), but in some stations since 2011. We now have a dataset to determine how light pollution is affecting astronomical observatories. A WEB portal was created to offer different downloads from these NSB data. We present the results of some elaborations for the 2018 dataset (statistics, histograms, annual and cumulative plots) for seven monitoring sites. For Ekar and Pennar sites, we also present the NSB monthly trend from 2014 until the time of the study. We purchased a reflex camera with a fish eye lens, appropriately calibrated with the software (SW) Sky Quality Camera, which allowed us to study ALAN using differential photometry. Here, we present our first results obtained by studying the night evolution of light pollution in the urban location of Padova.

## 1. Introduction

The night sky is never completely dark, even in the most isolated places there is a background glow resulting both from the natural component of terrestrial (such as auroral light) or extraterrestrial (such as zodiacal light) origin, and from the artificial component originating from human activities.

Nowadays, most of the population in industrialized nations live under skies where the artificial light at night (ALAN) exceeds natural light by thousands of times, with this being due to the luminous flux produced by artificial lighting, directly or indirectly sent towards the sky. The main effect of ALAN is the loss of perception of the Universe around us, as the increase in the brightness of the night sky due to artificial light prevents the vision of the starry sky, and also causes a strong environmental impact with negative effects on the biosphere, flora and fauna, and on human life, as well as influencing cultural aspects.

The Veneto region in the north-east of Italy (between 44 and 46 degrees North in latitude), is one of the most polluted regions in the world, especially with regard to light pollution [1], due to the presence of the contiguous industrial and commercial areas, and the continuous increase of lighting systems.

Its geographical conformation, which includes the Alps, the northern part of the Adriatic Sea, and a large plain, allows us to study the brightness of the night sky in relation to geographical location and altitude using instruments located in areas with significantly different environmental conditions and high population density.

Therefore, the aim of this study is to establish an effective and reliable network that allows monitoring over time of ALAN in different geographical areas, integrating the measurements carried out at astronomical professional observatories with those of amateur astronomy, thus realizing an excellent example of citizen science.

The monitoring of light pollution is expressly required by the law against the light pollution of the Veneto Region [2] that assigns this task to a committee coordinated by ARPA-Veneto.

In this work, we present the statistical analysis of the data collected by the monitoring network in 2018, and also study the temporal trend of brightness in two professional astronomical observatories in Asiago [3].

We also present our first results found with differential all-sky photometry with a commercial Digital Single Lens Reflex (DSLR) camera at a location of the monitoring network in urban area (Padova) [4].

## 2. Materials and Methods

Night sky brightness (NSB) is measured with a simple instrument named the Sky Quality Meter (SQM) [5] that consists of a specially calibrated sensor able to record light within a given field of view. The instrument is located in a fixed position and oriented towards the zenith. Due to their reliability, versatility, and cheapness, SQM devices have a great diffusion and are well suited to implement monitoring networks [6,7,8,9,10,11].

The NSB has a high degree of variability (not only from one night to another, but also within the same night) and includes contributions from natural sources, artificial diffused light (due to traffic, signs and streets lamps, public and private illumination, monument lights, advertising signs, etc.), and from astronomical sources (integrated light of stars and cosmic sources, diffused galactic light, zodiacal light, airglow, and auroral light).

The SQM is a low cost instrument, composed of a silicon photodiode (TAOS TSL237S) that, oriented towards the zenith and with an opening angle of about 20°, allows the recording of integrated brightness of the sky expressed in magnitude per second of square arc (mag·arcsec^−2^), with an uncertainty of the order of 10% [12].

The continuous monitoring of the NSB allows the recording of specific light pollution at each location, taking into account the variation of weather conditions, climate, and different moon phases, as well as the ability to study long-term trends.

In order to be able to analyse the data set recorded by SQM, we must ensuring its significance, which until now had been based exclusively on the instrumental calibration carried out by the manufacturer, and, therefore, validation of the data collected by each SQM cannot be ignored [12].

To ensure the traceability of the data to a reference standard, we carried out an inter-comparison test, based on a manual SQM “transfer” instrument, previously checked with a series of comparisons with reference instrumentation, in different light pollution conditions from the city to the darkest skies.

In these tests, the instrument confirmed the stability of the measurements over time, as well as the variation of the microclimatic conditions.

Given the complexity of the observations and the reduction of data, the observations at telescopes have been limited to a few nights distributed over the years, but are an important reference for the absolute calibration of the measurements of the SQMs that, although proven to be very stable and reliable, integrate a signal in a very wide spectral range that does not correspond exactly to the international astronomical standards of the blue (B) or visible (V) bands, in which the astronomical observations were historically made instead.

Transformations can be based on specific telescope observations or even using indications in the literature [5]. There is no simple transformation from the SQM system to the standard V system (visual) as there is dependence on the spectral distribution of the emission of the sky, which is dependent on the sources and the intensity of light pollution. For this reason, the SQM has been tested at different sites, at different levels of light pollution, and used in combination with the measurements of the sky in standard systems with some telescopes (La Silla, Chile; La Palma, Spain; Asiago, Italy). Brightness measurements with the telescope are reduced for accounting that light pollution brightness measurements are made "below the atmosphere".

The differences between data by SQM and those by the standard telescope system are of the order of 0.30–0.55 magnitudes, in accordance with an internal report by Cinzano [13]: additional differences are explained by the presence or absence of the Milky Way inside the field of view or by some bright sources that in the SQM are integrated with the sky.

Taking into account these sources, repeated measurements proved stable within a few percent, both between SQM and telescopes, and between different SQMs.

Recently, we have used the differential all-sky photometry with a commercial DSLR camera—Canon EOS 70D with a fish eye lens (Sigma with focal length 4.5 mm and aperture f 2.8). This technique allows the recording of information on the spatial distribution of light pollution in three spectral bands RGB [14,15,16,17].

The use of this technique in a stable and confined location allows for the recording images at regular intervals throughout the night. Here, we present our first results about the NSB evolution.

For the elaboration of the images we used the SW “Sky Quality Camera” (version 1.9.2, Euromix, Ljubljana, Slovenia), with a calibration carried out by the manufacturer of the SW [14].

## 3. Results and Discussion

The characteristics of the Veneto NSB monitoring network are presented in Table 1.

### 3.1. Veneto’s SQM Network and SQMWEB Portal

The NSB data were measured by the SQMs every 5 min and were recorded on a text file (ASCII) created by the same instrument. These files were then transferred (passing through some File Transfer Protocol servers), checked, processed, and sent to the database where they were stored. From the early hours of the morning, the NSB data were available on the "Live Data" page of ARPA-Veneto [18], where they were visible together with the data of the two previous nights.

Measurements of all SQMs were stored in a unique Oracle10g database owned by ARPA-Veneto. We now have more than 1,680,000 entries in the main table containing brightness data (for a few SQM we have data since 2011) collected in more than 13,000 nights. The data are available for users through a Drupal 7 portal named SQMWEB. This web portal includes some pages with different functionality: a simple reading page with nightly data from a single location/single date with calculation of the lunar phase, mean and median values of NSB in the astronomic night, slopes of brightness curves in different time intervals, sunset and sunrise hours, and types of night (cloudy or not). Furthermore, there are some statistic tools to calculate histograms of absolute frequencies, “hourglass” diagrams, “jellyfish” plots, diagrams of NSB monthly and annual trends, among other items. A page of download is also enabled with a filtering for location, time period, moon phase, or sky conditions and it gives a jellyfish plot and a histogram of brightness. This portal is still evolving, and we have used it experimentally to download the data for this research.

### 3.2. Statistical Analysis

Table 2 shows the data used (interval 12–22 mag_SQM_·arcsec^−2^) and the results of the statistical analysis, in which the full width at half maximum (FWHM) is referred to the peak of the histogram, only of the values of the new moon nights without cloud cover.

**Figure 1:**
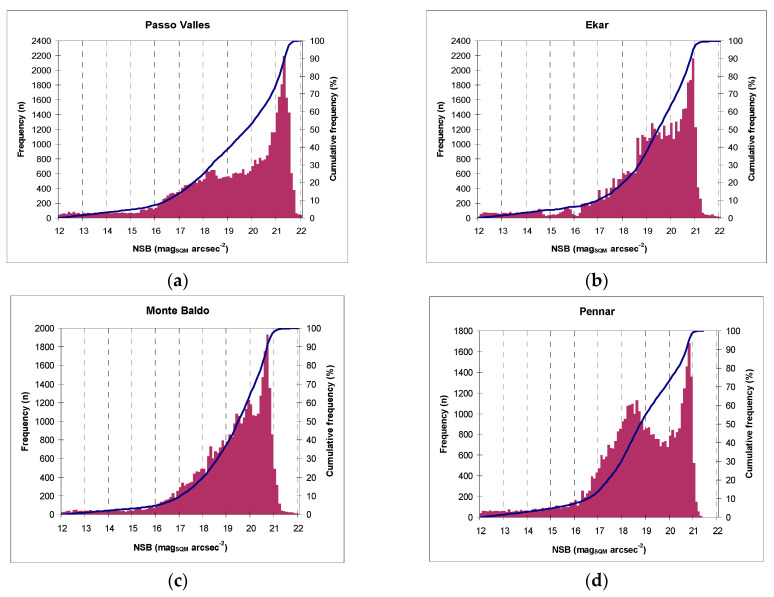

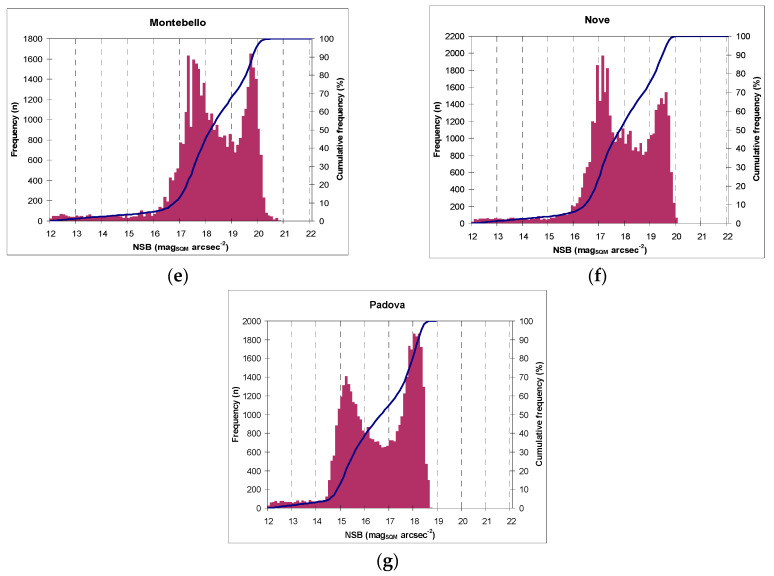
Histograms of absolute frequencies of NSB recorded during 2018 in the following locations: (**a**) Passo Valles, (**b**) Cima Ekar, (**c**) MonteBaldo, (**d**) Pennar, (**e**) Montebello, (**f**) Nove, (**g**) Padova.

NSB histogram plots for 2018 are presented for all network monitoring stations (Figure 1) in the interval brightness 12–22 mag_SQM_·arcsec^−2^. In addition, we also present the cumulative frequency of NSB values (blue curve). These figures were obtained from the entire dataset, including nights with clouds and moonlight.

It is noticeable that the distribution of absolute frequencies of brightness (Figure 1) has a characteristic shape depending on the type of station. In particular, histograms of urban and suburban stations are clearly bimodal, with a maximum at darker brightness values that corresponds to moonless and cloudless nights, and the brightest peak that includes both nights with the presence of the moon and those with a cloud cover.

The width of both the brightness peaks changed according to the effects of the cloudiness, but that at high magnitudes depended on the light pollution also present in the area.

The positions of the peaks of the NSB distribution represent the typical brightness of the location, while the distance between them depends mainly on the amplification caused by the reflection of the cloud cover, although in darker mountain sites it mainly includes the contribution of the moonlight [19].

As altitude increased and SQM devices were located far away from the urban site, the ALAN decreased. In rural and mountain locations, the distribution lost its bimodality, which was due to two main factors: First, ALAN was lower and, consequently, even in the case of cloud cover, the sky remained darker (especially in the case of more remote stations, for example, Passo Valles, as the stop of natural light); Second, some extremely dark values were likely caused by peculiar phenomena, such the presence of low clouds, and nights when snow covered the sensor [9,19,20].

The width of the distribution around the darkest peak can be studied by selecting only moonless and cloudless nights ("flat" night curves where the difference between NSB values are less than 0.05 mag_SQM_·arcsec^−2^ for at least 6 h). The calculated FMHW was different between locations and it was consistent with what had already been discussed. The value in darker mountain locations was less than half that of plain locations, most likely due to the different atmospheric variability, including the presence of particulate matter. The peculiarity of the station of Monte Baldo (a mountain site but with a typical FWHM of plain location with the absence of bimodality) can be attributed in the first analysis to the meteorological characteristics of the site, located not too far from a large lake basin (Garda Lake).

**Figure 2:**
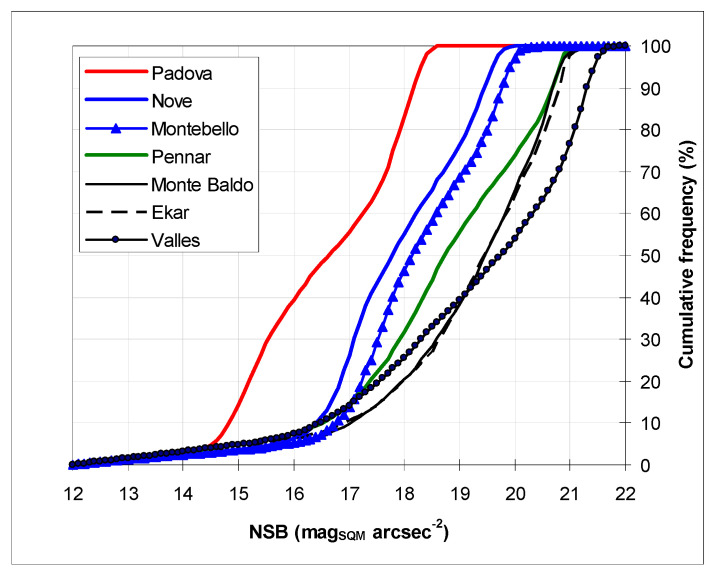
Cumulative frequencies of NSB in seven monitoring stations: red lines correspond to an urban station, blue to a suburban station, green to a rural station, and black to a mountain station.

In Figure 2 we show the cumulative frequency of NSB for each monitoring site—in urban and partially in suburban stations there was a saturation at smaller NSB values, and the distribution of NSB was due to cloud reflection, as already discussed above, confirming a previously published analysis by Barà [19].

### 3.3. Seasonal Variations of NSB

**Figure 3:**
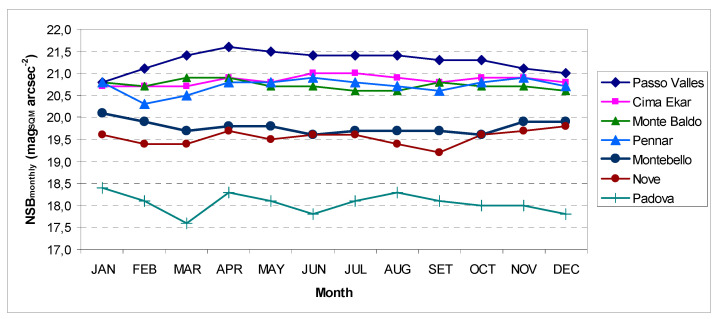
Monthly modal value of NSB during 2018.

In order to understand the seasonal variations of NSB in the different sites, we present the monthly modal values (i.e., the most frequent value) of NSB in Table 3 and Figure 3.

The NSB monthly evolutions were rather similar, but with some interesting specific characteristics.

In the winter months, there was an NSB minimum (more clear in Passo Valles), likely due to the Milky Way and snow cover of the ground [9]. The Milky Way disappears in spring when Monte Baldo and Passo Valles have both a maximum value of NSB.

In particular, the less polluted sites, such as Passo Valles, but also partly Ekar and Pennar, suffer more from local lighting, and for this reason we see a deterioration of the sky from November to February coinciding with the tourist period for skiing and holidays, whereas spring is the tourist off-season and, therefore, it recorded the darkest sky.

It is noticeable that the highest seasonal variability is in the urban and suburban sites, probably due to great but similar seasonal variability of cloud coverage, meteorological (humidity, rain, and fog), and environmental conditions (particularly air pollution) [7,21]. In the Padova site, a minimum value in December could be due to Christmas illuminations also, and a maximum value in August due to traffic decreasing for the summer holydays [22].

### 3.4. Nocturnal Development of NSB

**Figure 4:**
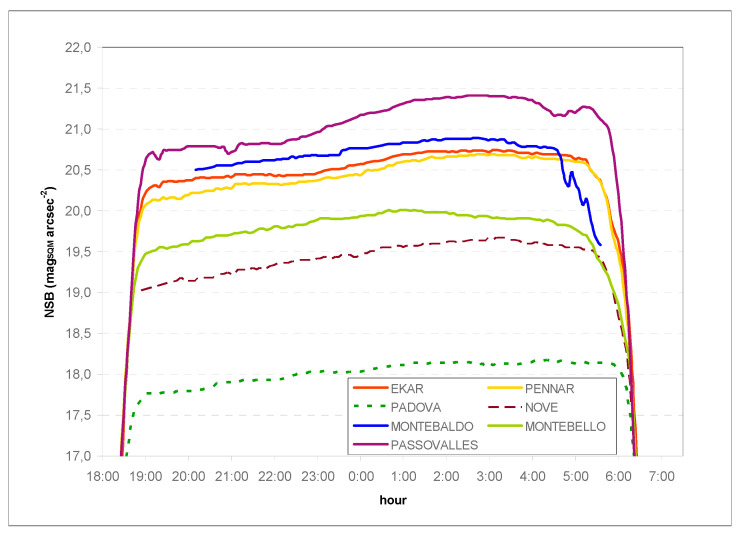
NSB data from 14 February 2018 to 15 February 2018.

Figure 4 shows the NSB recorded in all locations in a clear and moonless night on 14 February 2018 and 15 February 2018. The NSB hourly increase rate was calculated in the period of 21:00–01:00, and is shown in Table 4.

The decrease in NSB during the night was confirmed—the variation in traffic, reduction in luminous flux of street lighting, and the switching off of part of advertising signs and of private illumination caused the NSB to come down [21,23]. In the urban site (Padova), there was a smaller decrease (the SQM device is located downtown), while it was similar in the rural and subrural locations, with a maximum value in Passo Valles, probably due to the influence of local lights in a situation of a very dark sky.

### 3.5. Long-Term Trends in the NSB

**Figure 5:**
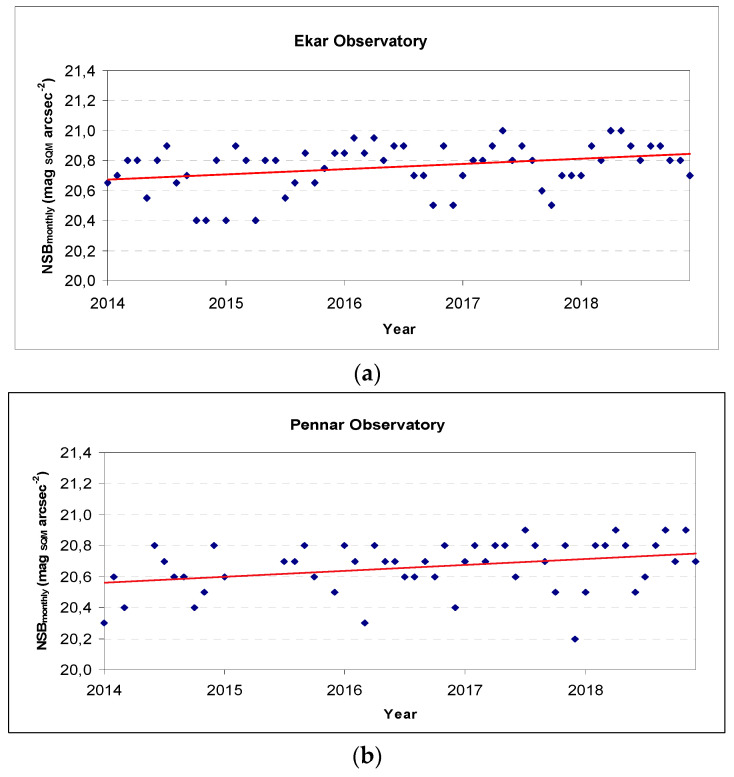
Ekar Observatory (**a**) and Pennar Observatory (**b**) monthly NSB trend.

In Figure 5 we represent the long-term trend of the monthly moonless modal value from 2014 to 2019 for the Ekar and Pennar sites (Asiago plateau), with its trend line (linear interpolation). These sites are of particular interest as the headquarters of professional astronomical observatories—given its location, the NSB recorded in these two places was influenced not only by local lights, but also by the lighting systems in the Padan Plain, whose edges lie just a few kilometres away.

In both sites (only 5 km away, but in different geographical and altitudes situations) there is a modal NSB value that progressively tends to rise, with a completely overlapping trend within the uncertainties (slope value of the linear regression equal to 0.0029 for Ekar and 0.0032 for Pennar).

This result seems to be in contrast with the increase in the number of public and private lighting installations in our region, both around the observatories and especially in the adjacent Padan Plain, roughly estimated for those years at 10% (unpublished ARPA-Veneto data).

It will be necessary to accurately study this crucial aspect in order to understand if it is a real decrease in light pollution, or whether related to changes in environmental parameters, such as humidity and particulate matter, and/or possible changes over the years of the spectral components, caused by the progressive replacement of discharge lamps with Light Emitting Diode LED solid-state lamps, which could cause an underestimation in the ALAN due to the bandwidth of the SQM used [24], or by a degradation of SQM instruments caused by a decrease of the transparency of the window.

We also did not correct the data for the decrease of solar activity. This could be an explanation for the observed decrease in the sky brightness but, since it is a small amount, further work is still needed to confirm this.

### 3.6. Differential Photometry with a Digital Camera

Differential photometry using a digital camera with a fisheye lens was used, not only to represent light pollution in all-sky, but also to investigate the temporal change of night sky brightness.

For this purpose, a time series of images were taken between 4 February 2019 at 19:00 to 5 February 2019 at 05:30 local time from the ARPA-Veneto headquarters in the city centre of Padova, in a typical urban environment, at regular intervals of 15 min (ISO sensitivity 1600; f 2.8; exposure time 15′).

**Figure 6:**
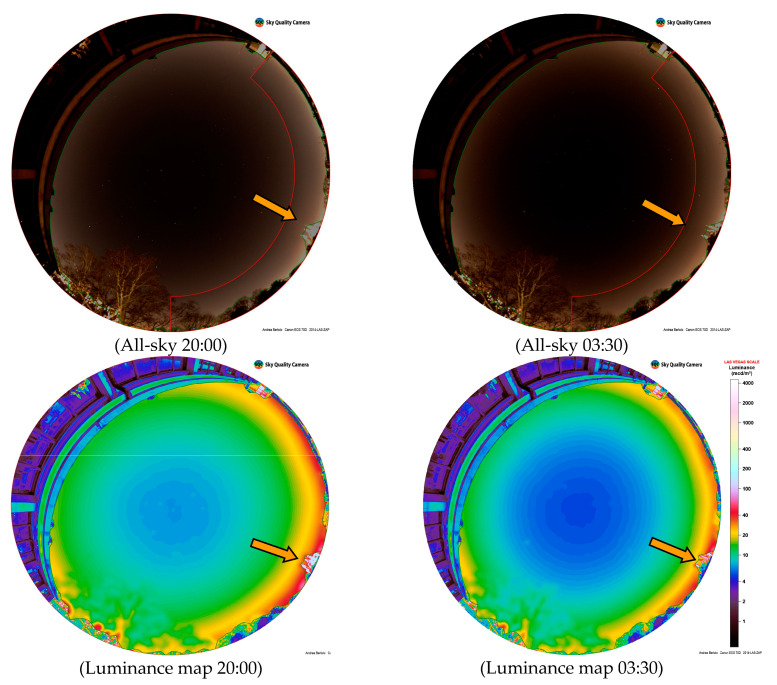

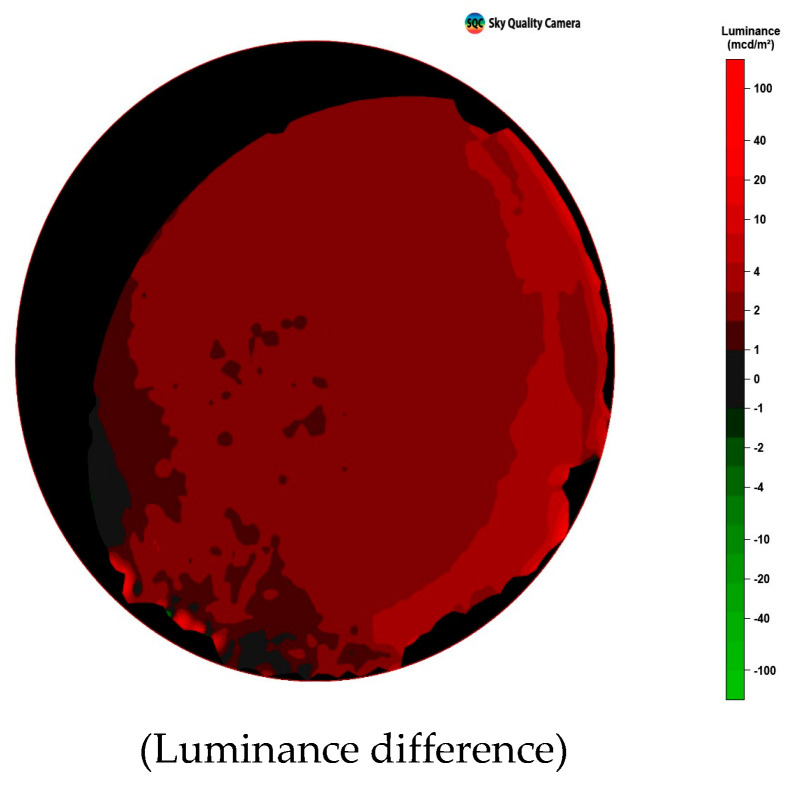
The upper row shows the all-sky RGB images at Padova, Italy, for a clear sky on 4 February 2019 at 20:00 local time and 5 February 2019 at 03:30 local time. The middle row shows luminance maps for the same time. The lower row shows the difference between luminance obtained from subtracting images. The orange arrow indicates Sant’Antonio ’s church.

In Figure 6 we present a clear night, the all-sky images, and the luminance maps obtained through the SW Sky Quality Camera that show the difference of ALAN during the night; the differential luminance map was obtained by subtracting the image with lower luminance (03:30) from the image with higher luminance (20:00). The subtracted map shows that the decrease in luminance occurs throughout whole sky, but is more pronounced in the city area visible on the horizon. Note the switch-off during the night of the lighting of the Sant’Antonio’s church, identified by the orange arrow in the figure.

The computing of the average luminance in the sector shown in red in Figure 6, which includes the part of the city visible up to the horizon not covered by buildings, are reported in Table 5 in comparison with the luminance measured with the SQM, and confirming the results.

**Figure 7:**
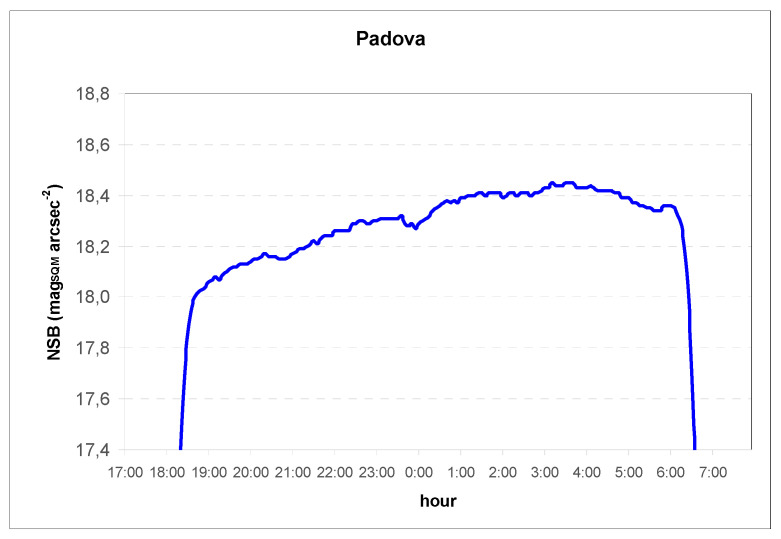
NSB at Padova by SQM for the night 2019/04/02–2019/05/02.

For the purpose of exhaustive information, the SQM values recorded by the monitoring unit at the same times are also shown, as well as the SQM trend recorded for the same night in Figure 7.

In order to make a comparison with the luminance value obtained from the luminance map, the NSB measured at zenith by SQM was converted into luminance at zenith using the approximate formula [15]:

L (cd/m^2^) ≅ 10.8 × 10^4^ × 10^−0.4 × L^_SQM_. (1)

The comparison between the luminance difference data shows that the decrease during the night was in absolute much smaller at the zenith in comparison with that of horizon, but fully comparable in relative terms, confirming that light pollution is dominated by artificial light dispersed from the city.

**Figure 8:**
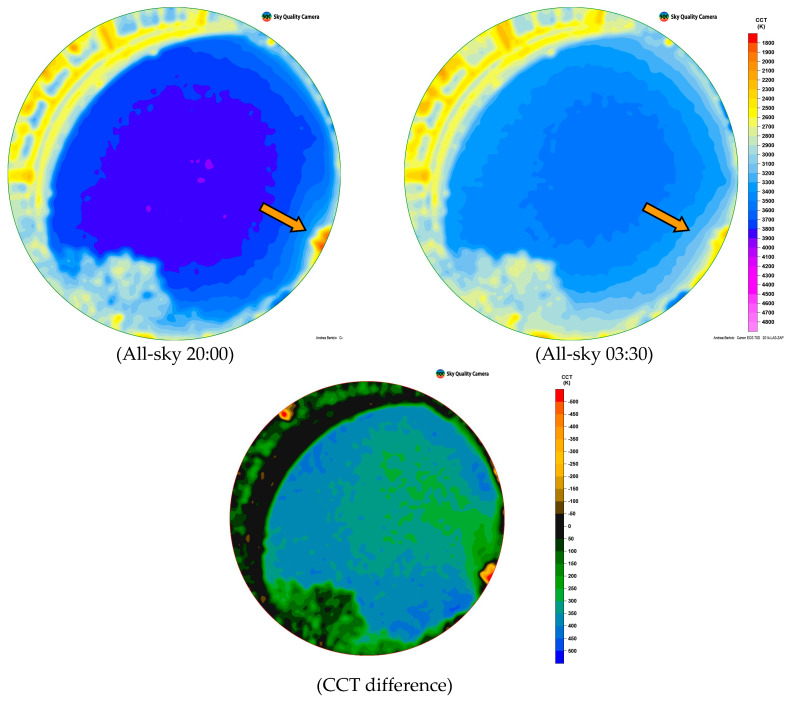
The upper row shows all-sky CCT images at Padova-Italy for a clear sky on 4 February 2019 at 20:00 local time and 5 February 2019 at 03:30 local time. The lower row shows the difference between CCT obtained from subtracting images. The orange arrow indicates Sant’Antonio s’ church.

We also presented for completeness (Figure 8) Colour Correlated Temperature (CCT) maps obtained through the SW Sky Quality Camera by the same images, and the differential CCT map obtained by subtracting the image at 03:30 from one at 20:00 [25].

The study by differential photometry of the night-time evolution of light pollution and correlation with the main ALAN sources will be the focus of our future analyses.

## 4. Conclusions

We present our first complete analysis of 2018 night sky brightness data recorded night after night by a Veneto regional SQM network. This dataset allows us to establish how the light pollution affects some observatories.

The continuous monitoring of the NSB allows for the recording of the specific light pollution of each location, taking into account the variation of weather conditions, climate, and different moon phases, as well as the ability to study long-term trends.

The annual night sky brightness modal value varied from 18.0 to 21.3 mag_SQM_ arcsec^−2^, confirming great variability of light pollution depending on the site position. The NSB absolute frequency distribution of the data generally appeared bimodal, but with significant differences in the widths of peaks and with relative distances.

In particular, the altitude of the site, its distance from main pollution sources, but also the different climatic variations, seem to play an important role.

The study of seasonal variations of NSB shows the greatest variability in urban and semi-urban locations, but also highlights the peculiar situations in the most remote stations.

The pooled study of the ALAN evolution in a single night in all the locations suggest several influencing factors, both natural (weather) and anthropogenic (air pollution, but also traffic and tourism).

In the Asiago plateau, the analysis of time trends over the last four years shows a decrease in light pollution, in contrast with the increase in regional lighting systems, which will need to be specifically investigated (in particular, the influence of spectral components and/or the degradation of SQM windows).

Finally, the use of all-sky images has been implemented: our first results indicate the great potential of this technique, used here to study the evolution of light pollution during the night. It shows that light pollution decreases at night time, but even at the zenith is due to the lights generated by the city.

In future papers, we will study the night sky brightness in correlation with meteorological and environmental factors (in particular the atmospheric particulate) and its night evolution in the network locations, also with the support of all-sky images.

## Figures and Tables

**Table 1:** Veneto’s Sky Quality Meter (SQM) Network.

Name	Latitude (N)	Longitude (E)	Altitude (m)	Type	Owner
Padova	45°24′09.7″	11°53′05.5″	12	Urban	ARPA-Veneto
Nove	45°42′32.92″	11°40′41.95″	77	Suburban	VenetoStellato
Montebello	45°28′7.40″	11°21′1.60″	212	Suburban	VenetoStellato
Pennar	45°51′57.52″	11°31′36.88″	1050	Rural	University of Padova
Monte Baldo	45°41′52.00″	10°51′32.00″	1208	Mountain	VenetoStellato
Cima Ekar	45°50′55.39″	11°34′8.34″	1366	Mountain	University of Padova
Passo Valles	46°20′19.79″	11°48′8.96″	2032	Mountain	ARPA-Veneto

**Table 2:** Data information and statistics night sky brightness (NSB) values.

Station	No. of Data	No. of Nights	Modal Value (mag_SQM_·arcsec^−2^)	Full Width at Half Maximum (FWHM) ^1^ (mag_SQM_·arcsec^−2^)
Padova	44,120	338	18.0	0.6
Nove	43,824	355	19.6	0.7
Montebello	39,199	318	19.7	0.6
Pennar Observatory	41,644	323	20.8	0.3
Monte Baldo	37,825	345	20.7	0.5
Ekar Observatory	44,480	342	20.9	0.2
Passo Valles	42,292	331	21.3	0.2

^1^ Moonless and cloudless nights only.

**Table 3:** Monthly modal NSB value (mag_SQM_ arcsec^−2^).

Station	Jan	Feb	Mar	Apr	May	Jun	Jul	Aug	Set	Oct	Nov	Dec
Passo Valles	20.8	21.1	21.4	21.6	21.5	21.4	21.4	21.4	21.3	21.3	21.1	21.0
Cima Ekar	20.7	20.7	20.7	20.9	20.8	21.0	21.0	20.9	20.8	20.9	20.9	20.8
Monte Baldo	20.8	20.7	20.9	20.9	20.7	20.7	20.6	20.6	20.8	20.7	20.7	20.6
Pennar	20.8	20.3	20.5	20.8	20.8	20.9	20.8	20.7	20.6	20.8	20.9	20.7
Montebello	20.1	19.9	19.7	19.8	19.8	19.6	19.7	19.7	19.7	19.6	19.9	19.9
Nove	19.6	19.4	19.4	19.7	19.5	19.6	19.6	19.4	19.2	19.6	19.7	19.8
Padova	18.4	18.1	17.6	18.3	18.1	17.8	18.1	18.3	18.1	18.0	18.0	17.8

**Table 4:** Hourly increase rate of NSB between 21:00 and 01:00.

Station	Hourly Increase Rate (mag/hour)
Passo Valles	0.148
Cima Ekar	0.070
Monte Baldo	0.073
Pennar	0.083
Montebello	0.075
Nove	0.080
Padova	0.053

**Table 5:** Average luminance in red sector of Figure 6, compared with the zenith NSB from Figure 7 on the nights of 4 February 2019 and 5 February 2019.

Data Source	2019/04/02 20:00	2019/05/02 03:30	Δ (mcd/m^2^)	Ratio
Luminance (mcd/m^2^)	22.78	19.29	3.49	1.18
SQM (mag_SQM_ arsec^−2^)	18.26	18.43	0.78 ^1^	1.19

^1^ Calculated by Equation (1).
